# Personalized dentistry: Enhancing outcomes through patient-centered innovation

**DOI:** 10.6026/973206300222470

**Published:** 2026-04-30

**Authors:** Aarushi Sharma, Anjali Tuteja, Mili Patel, Huma Tahir, Akash Daddanala, Aditi Pustake

**Affiliations:** 1 Department of Dentistry, Genesis Institute of Dental Sciences and Research, Ferozpur, Punjab, India; 2 Department of Dentistry, Goenka Research Institute of Dental Science, Gandhinagar, India; 3 Department of Dentistry, University College of Medicine and Dentistry, Pakistan; 4 Department of Dentistry, Sri Sai College of Dental Surgery, Telangana, India; 5 Department of Dentistry, Sharad Pawar Dental College and Hospital, Sawangi (Megha), Wardha, Maharashtra, India

**Keywords:** Personalized dentistry, genomics, salivary biomarker, artificial intelligence (AI), precision oral health care

## Abstract

Current dental practice relies on generalized treatment protocols that inadequately address individual genetic, biological and environmental variation, limiting precision in the prevention and management of oral and craniofacial diseases. Intricate interactions among these factors drive disease development, highlighting the need for more individualized approaches. Dentistry is moving toward personalized care through advances in digital technologies, salivary diagnostics and genomics. Therefore, it is of interest to describe the development and reach of personalized dentistry, with a focus on customized prevention and treatment based on genetic, lifestyle and clinical factors. With the use of biomarkers and patient-specific data, conditions such as caries and periodontal disease can be diagnosed earlier and managed more precisely. While these innovations promise better outcomes, challenges remain in ensuring data privacy and affordability.

## Background:

Most oral, dental and craniofacial diseases and disorders, such as dental caries, periodontal diseases, oral and pharyngeal cancers, chronic orofacial pain and cleft lip or cleft palate, arise from a complex interaction of genetic, biological, behavioral and environmental factors [1]. As our understanding of disease pathways, genomic interactions and novel biomarkers of oral conditions continues to grow, so does the potential to use high-throughput omics methods to assess risk, prevent disease and help with treatment [1]. This method, also known as genomic medicine, precision medicine, or personalized medicine, emphasizes how unique each patient's disease risk profile is, based on a confluence of their environmental, lifestyle and genetic characteristics [2]. While individual aspects of personalized dentistry have been explored in specialty-specific literature, a consolidated overview spanning diagnostics, prevention and treatment planning across all major dental specialties remains lacking. Therefore, it is of interest to review addresses that gap by describing the evolution, current evidence and clinical relevance of personalized dentistry, with the aim of providing practitioners across specialties with an integrated understanding of where the field stands today.

## Personalized dentistry (Evolution and scope):

A growing number of patients, physicians and scientists are supporting a personalized strategy that places equal emphasis on preventing disease and tailoring treatment after diagnosis [2]. People can receive treatments that are customized to their own distinct genetic, biological and social characteristics owing to this precise approach to healthcare, which differs from conventional one-size-fits-all strategies [3]. As new technologies and scientific discoveries enable more personalized approaches to patient care, modern dentistry is undergoing a significant transformation. Precision medicine, artificial intelligence (AI) and genomics are combining to provide more individualized and efficient dental care. These advancements are helping to change the way that various oral health conditions are diagnosed, treated and managed [4]. Concurrent advancement in nanotechnology is making it possible to create implantable oral bio devices that allow for real-time health monitoring and personalized drug delivery. Personalized pain management techniques may be enhanced by combining these technologies with new knowledge about the genetic and molecular causes of dental and orofacial pain, as well as by utilizing support systems that examine patient-specific data [3]. The development of advanced artificial intelligence (AI) technologies and improved data analytics techniques is anticipated to hasten the advancement of personalized dental medicine, even though it has not yet reached its full potential. These innovations can offer a more comprehensive understanding of their complex relationships by integrating data from the individual, clinical and system levels. This kind of integration could help the field move toward P4 dentistry, which stands for more individualized, predictive, preventive and participatory dentistry [4].

## Role of patient history and lifestyle in personalised care:

Each person's unique oral microbiome and individual factors require personalized oral care. Dentists consider various factors to create targeted plans for the prevention and management of oral diseases. Recording a patient's history has several important goals, such as obtaining the correct diagnosis, talking to the patient, educating them about their condition, making treatment plans, assessing the prognosis and taking care of the patient. In dentistry, it's very important to get a complete patient history and to be able to communicate well. Dentists can learn about a person's overall health and find risk factors by asking about their medical, dental and social history [5]. The patient's medical history, such as current conditions, medications and allergies, helps identify possible risks and factors when getting dental care. We can determine the health of teeth and gums, also the kind of treatment they require by their dental history, which includes previous procedures and issues with oral health. Additionally, understanding their lifestyle and social history enables us to develop prevention and care plans that are specifically tailored to them [5]. Systemic and local risk factors are the two categories of risk factors for various conditions. Local risk factors can be further divided into acquired and anatomical factors. Acquired local risk factors include plaque, calculus and poorly contoured restorations. Anatomical local risk factors include factors like enamel pearls, tooth crowding and furcation involvement. Systemic factors comprise multiple factors like diseases, dietary habits, genetics, age, smoking and socioeconomic status [6]. Preventive strategies for early childhood caries are informed by a comprehensive diet history that takes into account both individual and environmental factors, such as genetics and diet and consumption of sugary and acidic beverages. In addition to enabling customized treatment plans and caries prevention advice, this aids in identifying oral health risks. The risk of periodontal disease and dental cavities is increased by smoking and other lifestyle choices. By identifying high-risk individuals and providing individualized treatment, these conditions can be prevented and managed [7]. Oral symptoms like swelling and lesions can be caused by diseases like herpes, TB and leukemia. Because the mouth can harbor pathogens that increase the risk of disease, these conditions can occasionally manifest oral symptoms before other symptoms do [6]. Numerous systemic diseases, such as diabetes, lung disease, cardiovascular disease and pregnancy complications, are closely associated with periodontitis. The fact that poorly managed diabetes raises the risk of oral complications like periodontitis, oral candidiasis and accelerated periodontal bone loss emphasizes the importance of diabetes management for oral health [7]. Patients on long-term corticosteroids may need additional steroids prior to specific dental procedures in order to avoid adrenal crisis from adrenal insufficiency. Understanding the particular needs, emotional aspects and potential care barriers of older adults and people with special needs is essential when providing dental care. By increasing patient cooperation and adherence to treatment, effective strategies like non-invasive behavioral techniques can lessen the need for sedation or anesthesia [8]. Pregnant women require complete dental care during each trimester. A comprehensive strategy can ensure timely interventions, increase access to care and protect oral health during this critical period [9]. Barriers to effective oral care include limited patient knowledge, poor hygiene habits, dentist-patient dynamics, cultural factors and financial constraints [9]. Addressing underlying risk factors and promoting behavior change through practices like brushing, flossing and interdental cleaning to get rid of supragingival plaque are crucial for providing each patient with personalized care [8].

## Genetic, epigenetic and salivary biomarkers in oral health (Individual risk factors influencing susceptibility and care):

The advent of epigenetic, genetic and salivary diagnostics in dentistry has brought us closer to a precise, personalized, preventive and participatory approach to oral healthcare [10]. The early identification of individuals at risk of disease is an essential first step toward targeted preventive treatment. Structured caries risk assessment tools, designed for use by primary healthcare providers, have demonstrated particular value in the early identification of at-risk children, enabling timely and targeted preventive intervention [11]. Evidence indicates that inadequate maternal vitamin D levels during pregnancy may affect tooth calcification, predisposing to enamel hypoplasia and early childhood caries [12]. Genetic biomarkers show promising potential for use in diagnostics. One genetic marker discovered by a recent study is the amylase alpha-1 gene (AMY1), which codes for the salivary alpha-amylase enzyme. Larger studies are required for potential validation, but a higher copy number of this gene has been linked to a higher experience of smooth-surface caries [13]. Compared to blood, saliva is a sensitive, non-invasive, affordable and readily available method of detecting biomarkers, enabling real-time diagnostics and a lower risk of contamination. The two salivary biomarkers with the most clinical research are matrix metalloproteinases (MMPs) and interleukins (ILs). IL-1β, MMP-8, IL-6 and MMP-9 are the most frequently reported, followed by IL-8, TIMP-1, IL-10 and MMP-3 [14]. According to the findings of one study, patients with TMD myalgia had considerably higher salivary glutamate levels than controls, but lower salivary levels of BDNF (brain-derived neurotrophic factor) and NGF (nerve growth factor). However, depending on how the saliva was collected, the outcomes differed [15]. High levels of *C. gingivalis*, *P. melaninogenica* and *S. mitis* in saliva may serve as diagnostic markers for oral squamous cell carcinoma, according to the findings of another study [16]. Table 1 (see PDF) summarizes how salivary diagnostics can be used to differentiate between dental caries and periodontal disease-related dysbiosis [17]. A major challenge in the use of these biomarkers is to distinguish the normal, transient shifts in the oral microbiome from disease-associated changes. The full potential of personalized dentistry cannot be achieved without timely, non-invasive and easily accessible diagnostic tools during patient visits. This emphasizes the necessity of ongoing developments in biomarker and salivary diagnostic technologies [17].

## Personalised preventive strategies:

Caries remains the most prevalent disease in the world. A biofilm-mediated, diet-modulated, multifactorial, non-communicable, dynamic disease resulting in net mineral loss of dental hard tissues is the current definition of the condition [18]. Biofilm control is one of the most essential strategies and fundamental elements of the preventive management of dental caries [19]. For personalized caries management, the knowledge of lesion activity or progression, which is already difficult to establish through signs and symptoms alone, is not enough to inform targeted treatment. The analysis of the combination of factors involved in the disease aetiology and prognosis is also needed for a comprehensive diagnosis at the patient level [20]. Individual differences in caries susceptibility and progression rate are largely due to intricate interactions between protective and non-protective modifying factors as well as aetiological factors. The most reliable benefit for preventing the development of caries is fluoride, which can stop or even reverse the early stages of caries [20]. The availability and maintenance of fluoride in the oral cavity can be checked by thorough history taking. Such history taking will include information on habitual fluoride sources and concentration (i.e., water, toothpaste, rinses) and professional fluoride use [21]. Oral Hygiene Instructions (OHI) aim to increase knowledge, motivate self-efficacy and empower patients to plan healthy oral health behaviour and improve the skills of oral hygiene care. The OHI messages include the method, duration and time of tooth brushing and the use and choice of the toothbrush and toothpaste, which are delivered through verbal or written instructions [22]. A parallel randomised, single-centre study done in the UK demonstrated clinically significant improvements in bleeding on probing could be achieved in adults with mild to moderate gingivitis with the provision of OHI delivered using personalised video technology. Patient- specific personalised instructional videos were recorded by a qualified oral health educator, giving the participant OHI specific to their mouth, indicating which tooth surfaces needed more attention and how to clean these areas [23]. The time period between oral health examinations has been called the recall interval and it is part of the oral health examination and oral disease prevention process. The recall interval is based on information about individual risk factors as well as treatment response and oral disease history. During a recall interval appointment, oral health indices DMFT (decayed, missing, filled teeth), DT (decayed teeth), CPI (Community Periodontal Index, maximum value of individual was used) and the number of teeth in the oral cavity are assessed [24]. An observational, register-based study done in Finland showed a clear positive association between oral health indices and individualised recall intervals (IRI) for adults. It showed higher values of the DMFT and DT indices and CPI reduced the IRI [24].

## Personalized treatment planning across dental specialties:

Personalized dentistry acknowledges that every patient is unique. This is true for all specialties: what works for one individual might not work for another, as is discussed below.

## Periodontics:

Personalized periodontics is defined as the division of patients into distinct groups and the tailoring of clinical decisions, procedures and products to each patient. Most research in personalized dentistry conducted to date has predominantly focused on periodontology, likely because personalized workflows are easier to implement in periodontology than in other fields of dentistry, such as restorative and reconstructive dentistry [14]. Complete management of periodontal disease requires a recreational approach that addresses tobacco use, uncontrolled diabetes, obesity, cardiovascular disease and stress. Although bacteria are the cause of periodontal disease, the prognosis and clinical presentation are ultimately influenced by the inflammatory response of the individual as well as other modifying and predisposing factors [25]. Individual-specific genetic and environmental factors control the course of disease. Numerous lifestyle choices, such as tobacco use, uncontrolled diabetes, obesity, cardiovascular disease and psychological stress, must be considered in a personalized approach to managing periodontal disease [26]. Given that risk varies greatly from person to person, understanding these risk variables helps in the diagnosis and management of periodontitis pathobiology. In order to make well-informed clinical decisions regarding disease susceptibility, site-specific risk and treatment interventions, personalized medicine for periodontal diseases may soon use saliva to create subclinical profiles and identify and measure particular genotypes, phenotypes, putative pathogens, inflammatory markers and collagen-degradation biomarkers, as discussed previously [26].

## Prosthodontics and orthodontics:

Some patients may have an active social life and require aesthetics, while others may prioritize speech and chewing efficiency. By fusing enhanced diagnostic capabilities with material performance optimization, artificial intelligence is transforming customized treatment planning in prosthodontics [27]. To increase the accuracy of diagnosis and treatment, deep learning algorithms can analyze radiographs, CBCT images and intraoral scans to find early signs of occlusal wear. Diabetes, osteoporosis and bruxism are a few conditions that can affect prosthesis design, material selection and long-term care. Physicians can more accurately predict and treat the risks of temporomandibular disorder (TMD) by using AI-based motion tracking of the temporomandibular joint (TMJ) [27]. Comparably, AI-guided CBCT segmentation precisely determines bone density and volume, allowing for long-term stability and the proper placement of implants [28]. These data- driven insights ensure that prosthetic rehabilitation is tailored to the unique functional, structural and aesthetic requirements of each patient. Orthodontic treatments for children and adolescents are often timed to align with growth spurts, whereas those for adults are tailored to mature bone structures [29]. For example, Class III malocclusion is considered a monogenic dominant phenotype and it is also caused by the expression of certain genes that encode specific growth factors (Indian hedgehog homolog, parathyroid hormone-like hormone, insulin-like growth factor-1, vascular endothelial growth factor and harbor genes [chromosomal loci 1p36, 12q23 and 12q13], among others) [29]. AI-powered orthodontics takes customization to the next level, tailoring treatment regimens to each patient based on their unique dental anatomy and expected tooth movement. Planning takes into account how adjustments may impact facial balance, appearance and function, including chewing, in addition to tooth alignment. Some patients may prioritize speed, others aesthetics and some long-term stability-treatment is adjusted to align with these preferences. Clear aligner therapy uses AI technologies to design a series of aligners that apply exact amounts of force at each step of treatment, progressively shifting the teeth into their optimal positions as quickly as possible while reducing the patient's pain [30].

## Oral and maxillofacial surgery:

Virtual surgical planning (VSP) and computer-assisted design and manufacturing (CAD/CAM) are used to develop fitted surgical guides, cutting templates and customized hardware, assuring higher predictability, precision and efficiency in operations, including orthognathic surgery and reconstructive surgeries [31].

## Conservative dentistry:

In restorative dentistry, personalized treatment planning is a patient-centered approach that incorporates the functional, aesthetic and personal needs of the individual with a thorough clinical and diagnostic examination. It starts with a thorough examination, which includes a medical & dental history, caries and periodontal risk profile, occlusal analysis and radiographic or digital imaging, such as CBCT or intraoral scanning [32]. The dentist considers aspects such as remaining tooth structure, biting forces, parafunctional habits, systemic diseases and patient expectations. A meticulous and prioritized approach is created in light of these findings. This plan outlines the goals of the treatment, possible interventions, due dates and available materials while accounting for biological preservation, clinical evidence, patient preferences and financial constraints [32].

## Pediatric dentistry:

In pediatric dentistry, personalized treatment planning entails striking a balance between clinical findings and each child's distinct behavioral, developmental and risk profiles. The approach usually consists of three primary components: the child's caries risk, the available treatment options, the patient's compliance and behavioral state [33]. A thorough medical and dental history, caries risk assessment and clinical and radiographic examination laid the groundwork for selecting appropriate interventions from preventive sealants to stainless steel crowns or resin restorations while considering the child's oral habits and developmental stage. Using the International Caries Detection and Assessment System (ICDAS), dentists can design a completely personalized strategy that integrates precise cavity grading with the child's overall risk profile [34].

## Ethical and practical considerations in personalized dentistry (Data handling, access to care, cost, consent):

The development of customized dentistry raises a number of important issues that must be properly resolved to guarantee its responsible and efficient use. These include managing patient data, guaranteeing fair access to care, keeping costs under control and upholding informed consent.

## Data handling:

Privacy and security concerns are the main obstacles to the adoption of data-driven technologies in personalized dentistry. There are continuous discussions about broad consent, data sharing and privacy when weighing the advantages of data use for society against the defense of individual rights. Particularly concerning are cybersecurity flaws since they have the potential to reveal private medical data and undermine confidence in digital dental care. Systems need to be transparent, comprehensible and reliable in order for personalized dentistry to become more widely accepted [35]. Beyond security, there is still a chance of algorithmic bias and inaccurate forecasts, particularly when data is taken from small or non-representative samples. This risk in clinical decision-making is exacerbated by an over-reliance on automated guidance. Dental professionals still need stronger data literacy, making it crucial to incorporate statistics, data science and ethical data training into dental education, much like CAD-CAM was progressively adopted. Even though large-scale data analysis is now supported by quick improvements in computing power, storage and connectivity, access to these resources and high-quality datasets is unequal and frequently limited for commercial or proprietary reasons. Ensuring fair access and adopting shared standards and terminology are key to safe, consistent AI-driven personalized dentistry [35].

## Access to care:

As a reflection of larger health disparities, oral disease and insufficient dental needs are more common among older adults, people with disabilities, people with mental health disorders and people living in institutional settings [36]. Therefore, the development of personalized dentistry depends on expanding these groups' access to care. Teledentistry has been proposed as a potential solution by enabling remote consultations and collaborative care delivery; however, its use among vulnerable populations is still limited and there is little experimental evidence to support its effectiveness [36]. The increasing use of artificial intelligence in teledentistry holds promise for tailoring treatment to each patient's specific risk profile while boosting efficiency and cost-effectiveness through predictive analytics, diagnostic support and personalized monitoring. However, more excellent research is needed to validate these advantages and create a workable route to fair, individualized treatment [37].

## Cost:

The significant cost of personalized dentistry and medicine can restrict access and lead to disparities in availability. Making sure these innovations are accessible and affordable for all patients is crucial. Without this, individuals from lower-income groups may be unable to benefit from personalized care, existing health disparities may widen and scaling such healthcare solutions to broader populations will remain a major challenge [37].

## Consent:

Consent in personalized dentistry requires transparency, voluntariness and patient understanding, particularly with genetic testing and AI-driven tools [38, 39]. Important elements include defining the purpose of the test, possible outcomes such as incidental findings, care implications and anti-discrimination and data sharing policies [38]. Patients should be informed about how their data is used and given clear options to discontinue participation without compromising standard care. This is especially important as patient data is increasingly processed and reused in light of the growth of AI and digital platforms [39].

## Conclusion:

Personalized dentistry recognizes that each patient is uniquely shaped by their genes, habits, environment and overall health. Advances in genetics, salivary diagnostics and digital tools such as artificial intelligence are helping dentists understand these differences better and design treatments that truly fit each individual. The future of personalized dentistry depends on teamwork; it can go beyond technology to genuinely improve people's lives by assisting each patient in receiving care that is tailored to their needs, values and life story, provided that we maintain our focus on trust, transparency and fairness.
